# Association between Elevated TGA-IgA Titers and Older Age at Diagnosis with Absence of HBV Seroconversion in Celiac Children

**DOI:** 10.3390/vaccines9020101

**Published:** 2021-01-28

**Authors:** Chiara Maria Trovato, Monica Montuori, Andrea Sansone, Annalisa Morelli, Giusy Russo, Nicoletta Pietropaoli, Salvatore Oliva

**Affiliations:** 1 Pediatric Gastroenterology and Liver Unit, Maternal and Child Health Department, Sapienza University of Rome, 00161 Rome, Italy; chiaramaria.trovato@uniroma1.it (C.M.T.); m.montuori@policlinicoumberto1.it (M.M.); morelli.1578837@studenti.uniroma1.it (A.M.); giusy.russo@uniroma1.it (G.R.); nicoletta.pietropaoli@uniroma1.it (N.P.); 2 Chair of Endocrinology and Medical Sexology (ENDOSEX), Department of Systems Medicine, University of Rome Tor Vergata, 00161 Rome, Italy; andrea.sansone@uniroma2.it

**Keywords:** celiac disease, HBV seroconversion, children, anti-transglutaminase titers

## Abstract

Patients with celiac disease can have a low rate of protective hepatitis B (HBV) antibody titers after vaccination. We aimed to evaluate the HBV seroconversion in celiac disease (CD) children at the time of diagnosis as well as to identify the presence of possible predictive factors. Celiac disease children were prospectively enrolled and tested for antibodies against the S protein of HBV (HBsAg) at time of diagnosis between January 2009 and February 2020. Based on the serologic response to the vaccine, “responders” and “non-responders” were identified. Statistical analysis has been performed through R statistical software (3.5.1 version, R core Team) Of 96 CD children evaluated, 41.7% (n = 40) showed non-protective or absent antibody titers against HBV. Elevated IgA-antibodies against transglutaminase 2 (TGA-IgA) values and older age at diagnosis were associated with an absent seroconversion to HBV vaccine, while presenting symptoms were not significant. An elevated prevalence of absent seroconversion to HBV vaccine exists in this cohort of CD patients at the time of disease diagnosis. Elevated TGA-IgA titers and older age at diagnosis seem to negatively predict seroconversion. Further studies are needed to identify the real profile of “non-responders”, aiming to organize surveillance and eventual revaccination strategy.

## 1. Introduction

Celiac disease (CD) is an autoimmune systemic disorder elicited by gluten in genetically susceptible (HLA DQ2 and/or DQ8) subjects. It is characterized by the presence of a combination of gluten-dependent clinical manifestations or it may be discovered in asymptomatic individuals through screening methods (high-risk groups, first-degree relatives, and related diseases). CD-specific autoantibodies (anti-transglutaminase Ig-A TGA-IgA; anti-endomysium EMA), HLA-DQ2 or HLA-DQ8 haplotypes, and enteropathy are the lead actors of this disease [1]. Besides gastrointestinal symptoms, CD features several other manifestations [2], ranging from neurological [3] to autoimmune complications [4], or it can be completely silent [5].

In 2012, the European Society of Pediatric Gastroenterology, Hepatology and Nutrition (ESPGHAN) published new guidelines: among the recommendations included in these guidelines, the authors suggested evaluation of serology (i.e., TGA-IgA titers and EMA) before performing biopsy, and identified two different approaches to diagnosis, based on the symptoms reported by the child and on the genetic risk for CD [6]. These guidelines have been recently reviewed [7]: concerning diagnosis, the most important change was the omission of biopsy in all children with TGA-IgA values (≥10 times the upper limit of normal, ULN) and positive EMA, regardless of symptomatology.

Following the confirmation of diagnosis, the only available treatment is a life-long gluten-free diet (GFD)—by removing gluten from the diet, the patient also removes the initiating trigger for inflammation, therefore promoting mucosal healing [8]. Moreover, a strict GFD seems to improve dynamics of other autoimmunity when they are present at the moment of CD diagnosis [9,10].

While wheat is generally considered the chief culprit, the list of “usual suspects” for CD includes other gluten-containing cereals (such as barley, rye, spelt, and kamut). Any gluten free diet should be completely devoid of any and all of these cereals. On the other hand, pure oats [11] have been considered safe for celiac patients, both in children and adults, and also rice, corn, buckwheat, amaranth, millet, sorghum, and quinoa can be introduced in GFD as gluten-free cereals and pseudocereals. A strict GFD is often difficult to follow due to the risk of contamination—additionally, gravies and coatings used in food processing can often contain traces of gluten [12]. Nevertheless, such diets have often also been promoted by certain media in a sort of “celiac craze” in healthy subjects [13], following the wrong conception that removing gluten from any diet would yield positive effects on health.

Hepatitis B (HBV) is a viral illness responsible for a chronic infection that may cause cirrhosis and hepatocellular carcinoma. Through vaccination programs, the prevalence of HBV infection has remarkably decreased worldwide; however, 5% of the general population do not develop a response, while this rate appears to be even higher in specific groups (i.e., people with immunosuppression, autoimmune diseases, or dialysis-dependent) [14]. In CD, a lower rate of protective HBV antibodies was also reported, since about 40% of CD subjects can inadequately respond to the HBV vaccine [15].

HB vaccination represents the preferred strategy for the prevention of HBV infection, and it can be administered in young children as well as in adolescents or older subjects. Recombinant HB vaccines usually contain surface antigens (HBsAg) from HBV genotype A2, as they seem to have the highest efficacy. HBV vaccination has been suggested by the World Health Organization for all children in the world, including newborns, also based on cost-benefit analyses. Antibodies to hepatitis B surface antigen (anti-Hbs) induced by vaccination generally decline in the first 12 months following administration, then at a slower rate up to the point of undetectability over the course of several years—in order to be considered successful, reaching anti-Hbs titers of at least 10 mIU/mL is necessary [16], and a “booster” vaccination shot should be performed if anti-HBs fail to reach this threshold [17]. The causes of a failed response to vaccination are many, including the wrong site of administration, inadequate management and storage of the vaccine, as well as several health-risk behaviors, such as smoking and drug abuse, or infections and obesity, even more so in the adult population. Despite multiple studies, to date, the only two elements that seem related to a lack of HBV vaccine response in CD patients are: the dietary gluten introduction and the haplotype predisposing for CD development [18]. The dietary gluten has been hypothesized to have a role in the interaction between antigen presenting cells (APCs) showing CD-compatible HLAs and the HBV surface antigen. Due to a competition in the binding site, once bound to gluten APCs would have a lower affinity with the HBV antigen. This might lead to a decrease in the activation of T cells and, consequently, B cells, which would stunt the production of antibodies directed against the HBV surface antigen [19,20].

As known, the activation of gluten-reactive CD4+ T-cells in the gut, mediated by the DQ2 and DQ8 heterodimers, can be considered the first step in CD pathogenesis. The APCs express the disease-associated HLA-DQ molecules which specifically bind gluten-derived peptides modified by the enzyme tissue-transglutaminase (tTG); subsequently, the APCs present these peptides to intestinal T cells, eliciting a T response which triggers the production of antibodies and secretion of pro-inflammatory cytokines [21]. An abundance of gliadin can saturate the HLA molecules on APC, thus preventing vaccine HBsAg binding, which leads to the impaired T-cell activation and B-cell production [22]. An absent antibody response has also been associated with the presence of high-risk haplotypes predisposing to the development of CD, particularly to specific haplotypes, such as A*01-B*08 and HLA-B44 [23]. Indeed, HLA may represent the link between CD and lack of HBV vaccine response [24,25,26,27].

In addition to CD patients, protective immunity seems to not be reached in subjects with other autoimmune disease such as type 1 diabetes [28], with inflammatory bowel disease (IBD) [29], with human immunodeficiency virus (HIV), hepatitis C virus (HCV), and dialysis-dependent, end-stage renal disease (ESRD) [14], but immunological mechanisms are not identified in these specific populations.

To date, there is no clear consensus on the humoral response in CD children towards the HBV vaccine since available data remain quite isolated and heterogeneous.

Our study aim was to evaluate the prevalence of protective HBV antibody titers in CD children at the time of diagnosis and, secondarily, to identify predictive factors potentially determining the development of specific immunity, such as age, presence of symptoms and TGA titers.

## 2. Materials and Methods

We retrospectively enrolled pediatric CD patients diagnosed by biopsy at the Pediatric Gastroenterology and Hepatology Unit of the Policlinico Umberto I of Rome, Sapienza University, between January 2009 and February 2020. Enrolled patients were tested for antibodies against the S protein of HBV (HBsAg) at the time of CD diagnosis.

Patients with potential CD, IgA deficiency, other autoimmune disorders (type I diabetes mellitus, rheumatoid arthritis, Crohn’s disease, ulcerative colitis) and all subjects who had not undergone upper gastrointestinal (GI) endoscopy and/or HBV vaccination were excluded.

The TGA-IgA measurement was performed by using the Enzyme-Linked Immunosorbent Assay (ELISA) with a commercial kit (Eurospital, Trieste, Italy) and was normalized in upper limit of normal (ULN).

All subjects enrolled in the study underwent upper GI endoscopy under general anesthesia or deep sedation; multiple, targeted or random mucosal biopsies (at least four in duodenum and two in bulb) [30] were retrieved for each patient. Histological lesions were graded according to the Marsh–Oberhuber (MO) criteria [31].

The measurement of antibodies against the S protein of HBV (HBsAg) has been performed by sandwich-ELISA protocol [14]. A value ≥ 10 mIU/mL was considered protective.

The study population was divided into different groups based on the serological response to the HBV vaccine. Based on the serological response to the vaccination, we identified two groups: GROUP A (responders): CD children with protective titers against HBV (anti-HBsAg > 10 mIU/mL); GROUP B (non-responders): CD children with non-protective or absent titers against HBV (anti-HBsAg < 10 mIU/mL).

Children in group B did not have a protective title of antibodies at CD diagnosis, but it is impossible to state if they did not respond to vaccines or lost HBsAb protection.

We then measured differences in TGA levels, clinical presentation and age between the two groups, and performed multivariate regression analysis to further investigate the relationship between these variables. The study protocol has been approved by the Ethics Committee of Sapienza University Hospital.

### Statistical Analysis

Statistical analysis was performed using the statistical software R (3.5.1 version, R core Team) in order to run parametrical and non-parametrical statistical analysis.

More in detail, the two-tailed *t*-Test was used to measure differences in numerical variables between study groups; the Fisher test was used to measure differences in prevalence between study groups; and the logistic regression was used to measure how each parameter contributed to response to vaccine. The Wilcoxon rank sum test was used for comparison of median age between study groups. Statistical significance was set at *p* < 0.05.

## 3. Results

In our study cohort, 96 CD patients (62 F, median age 8.63 (IQR: 5.73–11.08) years; TGA-IgA mean 3.94 ± 3.65 ULN) were enrolled retrospectively. All patients had undergone testing for anti-transglutaminase antibody and anti-HbS antibody at the time of upper GI endoscopy, and all of them showed a mucosal damage compatible with CD (Marsh–Oberhuber classification 2–3). Characteristics of the study population are shown in Table 1.

GROUP A (responders) included 56 (58.3%) children (36 F, median age 6.93 (IQR: 4.57–9.84) years; mean TGA-IgA: 3.10 ± 2.90) with protective antibody values against HBV (anti-HBsAg > 10 mIU/mL).

GROUP B (non-responders): 40 (41.7%) children (26 F, median age 11.1 (IQR: 7.51–11.98) years; mean TGA-IgA: 5.10 ± 4.26) with non-protective or absent antibody values against HBV (anti-HBsAg < 10 mIU/mL).

### 3.1. Mean TGA-IgA and Serologic Response to the Vaccination

**Figure 1:**
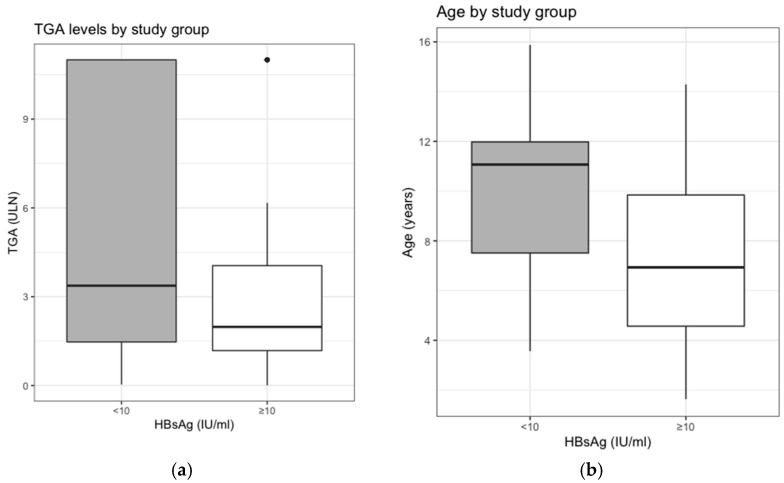
Differences between responders and non-responders in regards to (**a**) mean TGA-IgA titers (normalized in ULN) and (**b**) median ages.

The difference in mean TGA-IgA, normalized in ULN, between responders and non-responders was statistically significant (*p* = 0.012) (Figure 1a).

Group A (responders) has a mean TGA-IgA, normalized in ULN, of 3.10 ± 2.90, and Group B (non-responders) of 5.10 ± 4.26.

Therefore, subjects with an absent or scarce serological response to HBV vaccine have more elevated mean values of TGA-IgA in ULN.

### 3.2. Clinical Presentation and Serologic Response to the Vaccination

No statistical difference has been identified in the distribution of symptoms between the two groups.

In Group A (responders), symptoms compatible with CD were found in 49 (87.5%) patients and 7 were asymptomatic (screening for high-risk group or as part of population screening programs).

Group B (non-responders) included 33 (82.5%) symptomatic and 7 asymptomatic patients.

### 3.3. Median Age and Serologic Response to the Vaccination

The median age of our study population was 8.63 (IQR: 5.73–11.08) years.

Group A (responders) had a median age of 6.93 (IQR: 4.57–9.84) years and Group B (non-responders) had a median age of 11.1 (IQR: 7.51–11.98) years (Figure 1b).

A statistically significant difference (*p* < 0.001) has been found between the two groups, showing how subjects with a greater serological response to the HBV vaccine have a lower mean age compared to the *non-responders*.

### 3.4. Regression Analysis on the Response to the Vaccine

Regression analysis was performed in order to measure the effects of presenting symptoms, TGA-IgA values and age on the response to the vaccine. Elevated TGA-IgA values and age at diagnosis acted as risk factors for inadequate vaccine response (β = −1.67, SE = 0.73, *p* = 0.023 and β = −0.33, SE = 0.09, *p* < 0.001, respectively), whereas a non-significant effect of presenting symptoms was found (β = −0.96, SE = 0.75, *p* = 0.02). (Table 2). The resulting odds ratio is also reported in Table 2.

## 4. Discussion

The aim of our study was to evaluate the serological response to the HBV vaccination in a pediatric population at the time of CD diagnosis. According to previous data [22], 41.7% of our CD patients showed an inadequate response to the HBV vaccination, thus possibly not being protected. This rate of non-responders is higher than what has been reported in the general population (about 5%) [14].

Possible causes of this low vaccine response have not been clearly defined so far; however, HLA asset and dietary gluten seem to play a major role [18].

A meta-analysis study published in 2013, indeed, showed that specific HLA class II alleles (DRB1 and DQB1) seem to be associated with antibody response to HBV [23]; more in detail, DRB1 *03 (DRB1*0301), DRB1*04, DRB1*07, DRB1*1302 and DQB1*02 were associated with nonresponse to HBV vaccines, while DRB1*01, DRB1*1301, DRB1*15, DQB1*05 (DQB1*0501) and DQB1*06(DQB1*0602) were associated with a significant increase in antibody response to HBV vaccine. According to CD guidelines, genetic assessment was not mandatory for all subjects included in the study. Therefore, since the results of HLA testing were not available for all patients, we could not assess the association between specific HLA class alleles and antibody response vaccination.

By multiple regression analysis, we found a negative effect of elevated antibody titers (≥10 ULN) and age at diagnosis on the serological response to the HBV vaccine, while symptoms did not have a statistically significant impact in this regard.

**Figure 2:**
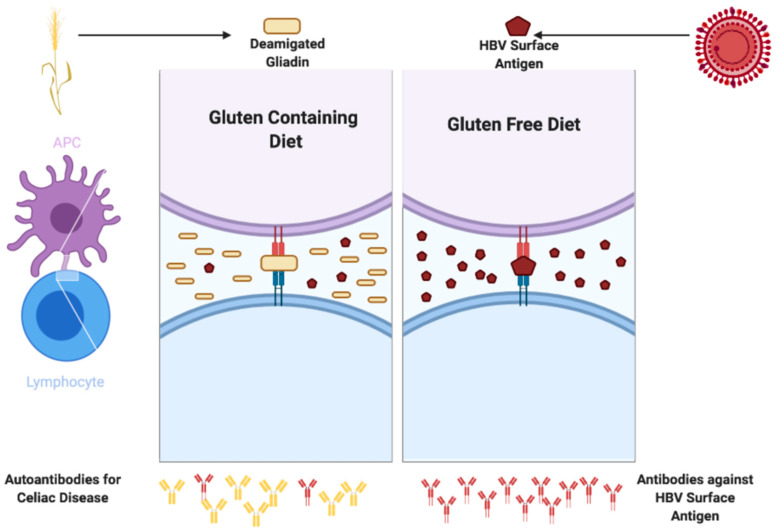
A possible mechanism through which the immune system may focus on the non-self antigen presented most commonly in these patients (dietary gluten) and may polarize its activity in this direction, rather than towards the hepatitis B (HBV) surface antigen, with massive production of TGA-IgA autoantibodies but suboptimal production of antibodies against the HBV surface antigens. (Figure created with Biorender).

To date, there are no studies in the literature that correlate the HBV vaccine antibody response to the CD antibody titers, and in particular to TGA-IgA titers. Our study shows that the production of a greater amount of TGA-IgA negatively correlates to the serologic vaccine response. This phenomenon might be explained by a competition between gluten and the HBV surface antigen. Thus, we may speculate that, in CD patients, the immune system may focus on the non-self antigen presented most commonly in these patients (dietary gluten) and may polarize its activity in this direction, rather than towards the HBV surface antigen, with massive production of TGA-IgA autoantibodies but suboptimal production of antibodies against the HBV surface antigens (Figure 2).

We also aimed to evaluate symptoms at diagnosis—differently from Ertekin’s results [32], we found no difference in the antibody production following HBV vaccination between different clinical presentations (over or asymptomatic). Furthermore, our data seem to be in accordance with what is described by Filippelli in 2016 [33]; in their study group, the distribution of the responders according to clinical features of celiac disease showed no statistically significant differences.

Symptoms seem to not affect the final autoantibody response against HBV, and they, as well known, did not correlate with TGA-IgA levels, histological damage or bone mineral density [34].

On the other hand, a correlation between serological response to vaccination and age at diagnosis has already been described [35]. Indeed, in the CD population, antibody levels for HBsAg appear to decrease faster than in a control population, and older CD individuals present lower antibody titers. Our results confirmed these data, with *non-responders* being significantly older (*p* < 0.001) compared to those with a positive serological response [36,37].

Furthermore, it seems that anti-HbS antibody titers gradually decrease in 15–50% of CD patients over 5–10 years, and finally become undetectable [38]. To date, however, there is no clear evidence concerning the possible development of an immunological memory that could protect against the virus, despite the negativity of the serological response to the vaccine. This hypothesis could be supported by the serological response that CD patients show to a booster dose of HBV vaccine, which has been demonstrated in multiple studies [22,39,40] and could be a useful approach in *non-responders* at the time of CD diagnosis. In addition, the response to a booster dose administered when patients are following a GFD could further prove the previously described competition mechanism—APCs would not be bound to gluten anymore and could therefore bind to the surface antigen of HBV, recognize it as “non-self”, and thus induce adaptive immunity and antibody production. This seems to be confirmed by results obtained by Nemes et al. [41], showing that the serological response correlated to a level of compliance to the GFD, with elevated percentages of seroconversion in highly compliant individuals. Based on these premises, investigating HBsAg titers could be a useful addition to the follow-up of CD patients [8]. Indeed, this hypothesis could be the principal reason for a secondary response to a booster dose after GFD. A multicentric study published in 2019 demonstrated that up to two-thirds of initial non-responders developed a serologic response following the administration of a single dose of booster vaccination [40].

Despite the presence of multiple studies in the literature suggesting benefits to the administration of a booster dose [22,39,40], to the present date there seem to be no unequivocal indications concerning the practical management of these patients. Multiple studies advocate for the creation of a specific vaccination schedule in this population [35,42], with attention to the type of vaccination (intramuscular or intradermal) because some data [43] indicate that intramuscular administration yields better results that the conventional one. Intradermal administration promotes a dendritic-cell-mediated immune response, rather than the T-cell-mediated response elicited by an intramuscular administration—this strategy could be beneficial in non-responders, with the added advantage of requiring less antigen [44].

Indeed, as suggested recently by Papadopoli et al. [45], it could be interesting to analyze the baseline anti-HBs levels, on which depends the response to a subsequent booster dose. As suggested, a booster dose of the HBV vaccine could be insufficient to reach an acceptable immunological response in subjects with undetectable anti-HBs titers at the baseline.

We recognize that this study may present several limitations, such as the retrospective, single-center nature of the study and the small sample-size; however, this the first study investigating whether different features of CD can affect response to vaccines. Another limitation of this study is the lack of analysis between serological HbsAg and HLA. Studies on large population samples are needed, however, in order to establish a possible re-vaccination timing and to clearly establish the non-responder CD patient profile.

## 5. Conclusions

In conclusion, our study confirmed an elevated rate (41.7%) of non-responders to the HBV vaccine among pediatric CD patients at the time of diagnosis. Elevated TGA-IgA titers (≥10 ULN) and older age at diagnosis seem to negatively predict seroconversion following HBV vaccination. Further studies will be necessary to identify the real profile of *non-responder* CD children in order to appropriately organize a surveillance program and/or identify possible strategies for revaccination.

## Figures and Tables

**Table 1:** Characteristics of the study population.

	Group A (anti HBs Ab > 10 mIU/mL)	Group B (anti HBs Ab < 10 mIU/mL)	*p*
Number	56	40	
Female	36	26	
Male	20	14	
Median Age	6.93 (IQR: 4.57–9.84)	11.1 (IQR: 7.51–11.98)	<0.001
TGA-IgA mean	3.10 ± 2.90	5.10 ± 4.26	0.012
TGA-IgA <10 ULN	51	28	
TGA-IgA >10 ULN	5	12	
Symptomatic children	49	33	n.s
Asymptomatic children	7	7	n.s

**Table 2:** Regression analysis on the response to the vaccine.

	β	Std. Error	OR [95% CI]	*p*
TGA titers (high vs. low)	−1.67	0.73	0.19 [0.04–0.81]	0.023
Symptoms (yes vs. no)	−0.96	0.75	0.38 [0.09–1.70]	0.20
Age (years)	−0.33	0.09	0.72 [0.60–0.86]	0.0002

## Data Availability

Data available on request due to restrictions eg privacy or ethical. The data presented in this study are available on request from the corresponding author. The data are not publicly available due to privacy.
